# Mesenchymal Stem/Stromal Cells Derived from Induced Pluripotent Stem Cells Support CD34^pos^ Hematopoietic Stem Cell Propagation and Suppress Inflammatory Reaction

**DOI:** 10.1155/2015/843058

**Published:** 2015-06-22

**Authors:** Mohsen Moslem, Irina Eberle, Iuliia Weber, Reinhard Henschler, Tobias Cantz

**Affiliations:** ^1^Department of Gastroenterology, Hepatology and Endocrinology, RG Translational Hepatology and Stem Cell Biology (OE 6817), Cluster-of-Excellence REBIRTH, Hannover Medical School, Carl-Neuberg-Street, 30625 Hannover, Germany; ^2^Department of Transfusion Medicine, Cell Therapy and Hemostaseology, Ludwig-Maximilian University Hospital, Max-Lebsche-Platz 32 A, 81377 Munich, Germany; ^3^DRK Institute of Transfusion Medicine and Immune Hematology, Frankfurt, Germany; ^4^Cell and Developmental Biology, Max Planck Institute for Molecular Biomedicine, Münster, Germany

## Abstract

Mesenchymal stem/stromal cells (MSCs) represent a promising cell source for research and therapeutic applications, but their restricted *ex vivo* propagation capabilities limit putative applications. Substantial self-renewing of stem cells can be achieved by reprogramming cells into induced pluripotent stem cells (iPSCs) that can be easily expanded as undifferentiated cells even in mass culture. Here, we investigated a differentiation protocol enabling the generation and selection of human iPSC-derived MSCs exhibiting relevant surface marker expression profiles (CD105 and CD73) and functional characteristics. We generated such iPSC-MSCs from fibroblasts and bone marrow MSCs utilizing two different reprogramming constructs. All such iPSC-MSCs exhibited the characteristics of normal bone marrow-derived (BM) MSCs. In direct comparison to BM-MSCs our iPSC-MSCs exhibited a similar surface marker expression profile but shorter doubling times without reaching senescence within 20 passages. Considering functional capabilities, iPSC-MSCs provided supportive feeder layer for CD34^+^ hematopoietic stem cells' self-renewal and colony forming capacities. Furthermore, iPSC-MSCs gained immunomodulatory function to suppress CD4^+^ cell proliferation, reduce proinflammatory cytokines in mixed lymphocyte reaction, and increase regulatory CD4^+^/CD69^+^/CD25^+^ T-lymphocyte population. In conclusion, we generated fully functional MSCs from various iPSC lines irrespective of their starting cell source or reprogramming factor composition and we suggest that such iPSC-MSCs allow repetitive cell applications for advanced therapeutic approaches.

## 1. Introduction

Regarding clinical stem cell applications, mesenchymal stem/stromal cells (MSCs) have been introduced as a favorable cell type, which can be maintained* ex vivo* and have the potential to regenerate mesodermal tissues such as cartilage, tendon, bone, and muscle in variety of skeletal diseases (for review see [1]). Furthermore, MSCs can support hematopoiesis [2, 3] and are able to modulate inflammatory reactions by dynamic interplay with the innate and adaptive immune systems [4–6]. However, the limited proliferation capability of MSCs during long-term culture leading to cellular senescence after 8–10 passages challenges the generation of large-scale cell yields, which would be essential for repetitive therapeutic applications. In principal, such needs would be met by pluripotent stem cells exhibiting an unlimited proliferation capacity and that can be generated from patients' samples via reprogramming of somatic cells into induced pluripotent stem cells (iPSCs) [7–10]. Such human iPSCs are responsive to differentiation stimuli during* in vitro* cultivation and in the recent past the generation of iPSC-derived MSCs (iPSC-MSCs) was described and it was demonstrated that iPSC-MSCs displayed comparable antigen profile and differentiation capability to bone marrow MSCs (BM-MSCs) and exhibited considerable functional properties [11–16]. Moreover, there is convincing evidence that iPSC-MSCs with higher expansion capacities can be transplanted in many degenerative diseases resulting in similar outcomes as BM-MSCs [13, 15, 17]. Increasing evidence, however, indicates that MSCs from different origins are heterogeneous populations exhibiting variable gene expression patterns [18, 19], presenting different surface markers [20], or showing reduced proliferation potential and differentiation capacities [21–23].

Furthermore, a successful approach of iPSC-based therapeutic cell applications in regenerative medicine depends on the ability to set up an efficient differentiation protocol resulting in a desired cell population with a high purity. Most importantly, harmful contaminations of undifferentiated pluripotent stem cells must be avoided, to exclude the risk of teratoma formation. Therefore, the robust generation of a homogenous iPSC-MSC population with cellular characteristics identical to bona fide MSCs and similar or even enhanced functional capabilities such as proliferation, hematopoietic support, and anti-inflammatory responses need further attention. Here, we exploited the differentiation potential of three iPSC lines generated from fibroblast or primary MSCs with Yamanaka reprogramming factors [10], namely, Oct4, Sox2, Klf4, and c-Myc (OSKM) or Thomson factors [7], namely, Oct4, Sox2, Nanog, and Lin28 (OSNL). Upon MSC differentiation we applied lentiviral selection constructs carrying CD105- and CD73-promoter driven fluorescent reporter and Neomycin/Puromycin-resistance-transgenes to enrich the bulk differentiation for fully differentiated MSCs. Next, we explored the antigen profile, differentiation potential, proliferation capacity, hematopoietic support, and immune-suppression potential in regulation of lymphocyte proliferation, proinflammatory cytokine secretions, and activation markers of such iPSC-MSCs in direct comparison to bone marrow MSCs (BM-MSCs) from three different donors (LM02, LM05, and LM06).

## 2. Material and Methods 

### 2.1. Human iPS Cell Culture

Human fetal liver fibroblast (FLF) iPS cells were provided from in-house supplies using transduction via lentiviral reprogramming factors Oct4, Sox2, Klf4, and c-Myc (OSKM) [24] and Oct4, Sox2, Nanog, and Lin28 (OSNL) [25]. Human iPSCs were cultured on irradiated mouse embryonic fibroblasts (MEF) in a humidified incubator at 37°C and 5% CO_2_ in medium containing DMEM/F-12, 20% knockout serum replacement (Life Technologies), 20 ng/mL human recombinant basic fibroblast growth factor (bFGF, provided from Leibniz University Hannover), 0.1 mM *β*-mercaptoethanol (Life Technologies), 1 mM L-glutamine, 1% nonessential amino acids, and 1% penicillin/streptomycin (all from Sigma-Aldrich).

Media were changed daily and cells were split weekly by dissociation with 200 U/mL of collagenase IV (Life Technologies) and cells were plated on Matrigel-coated plates in medium supplemented with 40 ng/mL bFGF for further differentiation.

### 2.2. Derivation and Enrichment of Human MSC-Like Cells

For triggering iPSC differentiation toward MSC-like cells, human iPSC colonies grown on Matrigel (Corning) were maintained with MSC induction media consisting of DMEM (low-glucose, Sigma-Aldrich), 10% defined fetal bovine serum (FBS, STEMCELL Technologies), 1% nonessential amino acids, 1% penicillin-streptomycin, and 2 ng/mL human recombinant bFGF for 7 days. Next, cells were treated with collagenase IV for 3 min at 37°C, dissociated by glass beads and gentle pipetting, and then passed through 40 mm cell strainers (Fisher Scientific). Single cells were seeded onto gelatin-coated plates at 1 × 10^4^ cells/cm^2^ in MSC media.

To facilitate enrichment and screening of MSCs during the standard differentiation protocol of iPSCs into MSCs, a combination of two positive markers, namely, CD73 and CD105 (which are consistently expressed in MSCs), was chosen to produce MSC-specific selection vectors. The promoter regions for CD73 and CD105 were amplified and ligated into the corresponding lentiviral backbone; the CD105 promoter into pRRL-Puro-IRES-GFP; and the CD73 promoter into pRRL-neo-IRES-dTom. (Lentiviral backbones provided in-house based on lentiviral constructs from Axel Schambach laboratory). Cells were selected by 500 *μ*g/mL G-418 and 4 *μ*g/mL Puromycin-dihydrochloride (both from Sigma-Aldrich) in culture media for 2 weeks until the untransfected cells were killed. Functionality of the vectors confirmed fluorochrome expression in transduced cells by fluorescent microscope afterwards.

Double-positive MSCs population transduced with pRRL-CD105-Puro-IRES-GFP and pRRLCD73-neo-IRES-dTom was purified with the FACSAria II cell sorter (BD Bioscience). A total of 2 × 10^6^ sorted cells were immediately plated back into gelatin-coated plates to facilitate adherence. After 24 hours, fresh prewarmed MSCs medium was added and cells were allowed to expand and reach nearly 100% confluence. Cells were counted in different time points. Bone marrow MSCs were isolated in Frankfurt university hospital as previously described [26]. Shortly 10–30 mL of bone marrow was aspirated from femoral cavity of patients who needed hip joint replacement surgery after informed consent in accordance with the Declaration of Helsinki. After density gradient separation, the light density mononuclear cell fraction was seeded on T25 (TPP) tissue culture flasks in previously mentioned MSC media.

### 2.3. Lentiviral Vectors Production

HEK 293T cells were used for virus production. 3 × 10^6^ cells were seeded one day before transfection in 10 cm dishes (TPP) in DMEM (high glucose, Life Technologies) supplemented with 10% FBS and 1% penicillin/streptomycin and 1% L-glutamine. The next day, medium was exchanged with 8 mL DMEM supplemented with 25 *μ*M Chloroquine (Sigma-Aldrich). Plasmids encoding for lentiviral gag/pol (pCDNA3.GP.CCCC, 10 *μ*g), RSV-Rev (pRSV-Rev, 5 *μ*g), VSV-G (pMD2.G, 2 *μ*g), and packaging plasmid encoding for respective transgene into pRRL-Puro-IRES-GFP and pRRL-neo-IRES-dTom were mixed in 400 *μ*L of ddH_2_O and 100 *μ*L of 1.25 M CaCl_2_. The plasmids-CaCl_2_ mixture was added dropwise to 2xHBS and observed until precipitates became visible in phase-contrast microscope and then added to HEK cells. 6 hours later, medium was exchanged with 10 mL DMEM high glucose supplemented with 10% FBS and 1% penicillin/streptomycin and 1% L-glutamine. 48 hours later, supernatant was collected, passed through 0.45 *μ*m filters, and centrifuged at 14,000 ×g for 8 h. Virus pellet was resuspended in 200 *μ*L PBS (Sigma-Aldrich). Viral titers were determined by transduction of HEK 293T cells in serial dilutions and analysis of reporter gene expression by flow cytometry. Generally, titers were in the range of 1-2 × 10^7^ viral particles per mL.

### 2.4. Antigen Profiling by Flow Cytometry

To assess the immunophenotypic profile of BM-MSCs and iPSC-derived MSCs, single cell suspensions were prepared by trypsin digestion (Life Technologies) and washed with cold PBS containing 1% bovine serum albumin BSA (Merck Millipore). Next, 2 × 10^5^ cells were incubated for 30 minutes with the respective APC-conjugated monoclonal antibodies: CD73, CD90, CD105, CD45, CD34, and CD19 (all from BD Bioscience listed in Table 1), and subsequently resuspended in a density of 2 × 10^5^ cells per 200 *μ*L in cold PBS containing 1% BSA. Nonspecific fluorescence was determined by incubation of cell aliquots with isotype-matched monoclonal antibodies.

Samples were run on a FACS Calibur (BD Bioscience) cytometer using FACS Diva software. For each analysis, a minimum of 10,000 cells was assayed. Data was further processed using FlowJo Software (Tree Star).

### 2.5. Growth Kinetics

Human BMSCs from 3 different donors (LM02, LM05, and LM06) and 3 iPSC-MSCs lines were plated (2 × 10^4^ cells/well) onto 12-well plates in triplicate. Cells were harvested after 72 hours in each passage (10 passages for BM-MSCs and 15 passages for iPSC-MSCs). Cumulative population doublings were calculated using the formula: *x* = [log⁡10(NH) − log⁡10(*N*1)]/log⁡10(2) [27], where *N*1 is the inoculum cell number and NH the cell harvest number. To yield the cumulated doubling level, the population doubling for each passage was calculated and then added to the population doubling levels of the previous passages. The cultures were abandoned as soon as they showed a senescent phenotype when they ceased proliferation.

### 2.6. *In Vitro* Adipogenic, Chondrogenic, and Osteogenic Differentiation

Differentiation induction of iPSC-MSCs was carried out for 21 days in different differentiation media. Totally 10^4^ cells were seeded per well in six-well plates (TPP). To induce osteogenic differentiation, cells were cultured with MSC medium containing 1 *μ*M dexamethasone, 0.5 *μ*M ascorbic acid, and 10 mM b-glycerol phosphate (all from Sigma-Aldrich). For adipogenic induction, cells were cultured in MSC medium supplemented with 50 *μ*g/mL indomethacin (Sigma-Aldrich), 50 *μ*g/mL ascorbic acid, and 100 nM dexamethasone. For chondrogenic differentiation, iPSC-MSCs were centrifuged in 0.2 mL of medium at 500 g for 10 min in 15 mL Falcon tubes to form a pellet. The pellets were cultured in MSC medium supplemented with 0.01 *μ*M dexamethasone, 397 *μ*g/mL ascorbic acid-2-phosphate (Sigma-Aldrich), 1 mM sodium pyruvate (Sigma-Aldrich), 10 ng/mL transforming growth factor-*β*1 (TGF-*β*1, Life Technologies), and 1% insulin-transferrin-selenium (Life Technologies). Osteogenesis was assessed by alizarin red staining, adipogenesis by oil red staining, and chondrogenesis by alcian blue staining (all from Sigma-Aldrich).

### 2.7. Real Time qRT PCR

Total cellular RNA was isolated using TRIzol reagent (Life Technologies). Resultant RNA was subjected to DNase treatment and cDNA Synthesis Kit (Life Technologies) with random hexamers. Power SYBR Green Master Mix based qRT PCR assays were performed on the StepOne Plus Cycler (Applied Biosystems) using the standard settings. We collected samples from at least three independent experiments. Expression values of human peroxisome proliferator-activated receptor *γ* (PPAR*γ*), PPAR*α*, lipoprotein lipase (LPL), collagen type II (COL2), aggrecan (ACAN), osteocalcin (OCN), and alkaline phosphatase (ALP) normalized to expression of GAPDH. The primer sequences are listed in Table 2.

### 2.8. Coculture of CD34^+^ Progenitor Cells with iPSC-MSCs

Human iPSC-MSCs or BM-MSCs layers were grown until 80% confluency in six-well plates and then treated with mitomycine-C (Sigma-Aldrich) to prevent cell overgrowth. After 24 h, medium was removed and purified CD34^+^ cells (resuspended at 7.5 × 10^4^ cells per well) were then added in 2 mL of long-term culture medium containing *α*-minimal essential medium (Life Technologies) with 20% FBS, 1 *μ*mol/L hydrocortisone (Sigma-Aldrich), and 0.1 mM *β*-mercaptoethanol. The cocultures were incubated for 20 days medium exchange twice per week. Nonadherent viable cells were counted at the indicated time points (day 10 and day 20). CD-markers for hematopoietic differentiation were also determined after 8 days for CD34, CD45, CD-11b, and CD-14. Experiments were repeated three times.

### 2.9. *In Vitro* Progenitor Assays

Effects of human iPSC-MSCs or BM-MSCs on progenitor cells were analyzed using a colony forming cell assay. Human bone marrow CD34^+^ cells (2 × 10^6^) were obtained from Lonza and were plated in 2 mL of methylcellulose media (STEMCELL Technologies) with or without iPSC-MSCs and BMSCs. Colonies of >50 cells were scored after 4 and 8 days of incubation.

### 2.10. Assessment of CD4^+^ T-Lymphocyte Proliferation Response to iPSC-MSCs

Standard 5-day MLR cultures were set up with 5 × 10^4^ Mitomycin C–treated (Sigma-Aldrich) human peripheral blood mononuclear cells (PBMCs) as stimulators and 2 × 10^5^ human CD4^+^ T-cells (Lonza) in 96-well round-bottom plates in 200 *μ*L complete medium consisting RPMI 1640 (Life Technologies) supplemented with 0.1 mM *β*-mercaptoethanol, 10% FBS, GLUTAMAX I (Life Technologies), 100 U/mL penicillin, and 100 *μ*g/mL streptomycin in the presence or absence of iPSC-MSCs and BM-MSCs. For analyzing expression of CD69^+^ and CD25^+^ regulatory T-cell population, 10^6^ responder cells were mixed with 2.5 × 10^5^ stimulator PMNCs in presence or absence of 2 × 10^5^ iPSC-MSCs or BM-MSCs. MLRs were performed on a layer of confluent Mitomycine C treated MSCs seeded one day before. Proliferation was determined with BrdU ELISA assay (Roche) based on manufacturer instruction. IL-2 and IFN-*γ* concentration was determined in MSC/MLR coculture supernatants using a commercially available ELISA (BD Bioscience) according to manufacturer's instructions. CD25 and CD69 (BD Bioscience) expression on CD4^+^ cells were analyzed by flow cytometry.

## 3. Results and Discussion

### 3.1. Generation of MSCs from Human iPSCs with Spindle-Shape Morphology

**Figure 1:**
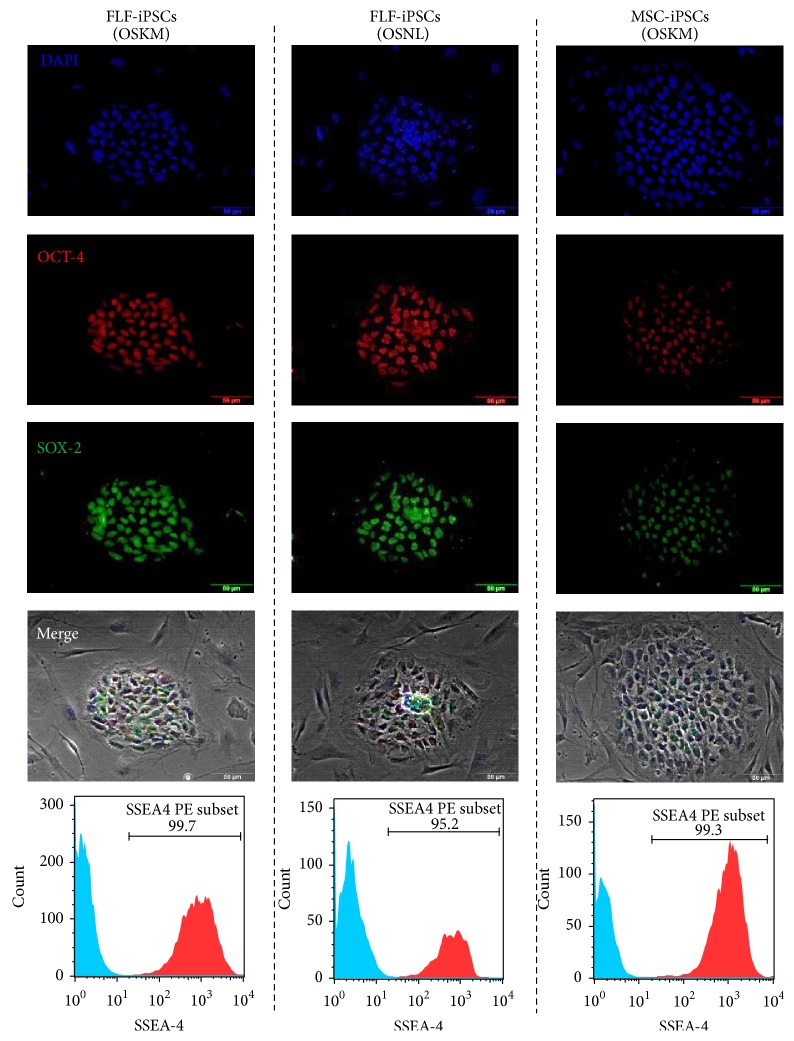
Generation and characterization iPS cells from human fetal liver fibroblasts (FLF) with Oct4, Sox2, Klf4, and c-Myc (OSKM) and Oct4, Sox2, Nanog, and Lin-28 (OSNL) and also from human bone marrow MSCs with OSKM using lentiviral vectors. iPSCs stained positive for human OCT4 and SOX2. DAPI was used to stain the nuclei and merged with phase-contrast. Expression of SSEA-4 is shown in histograms.

**Figure 2:**
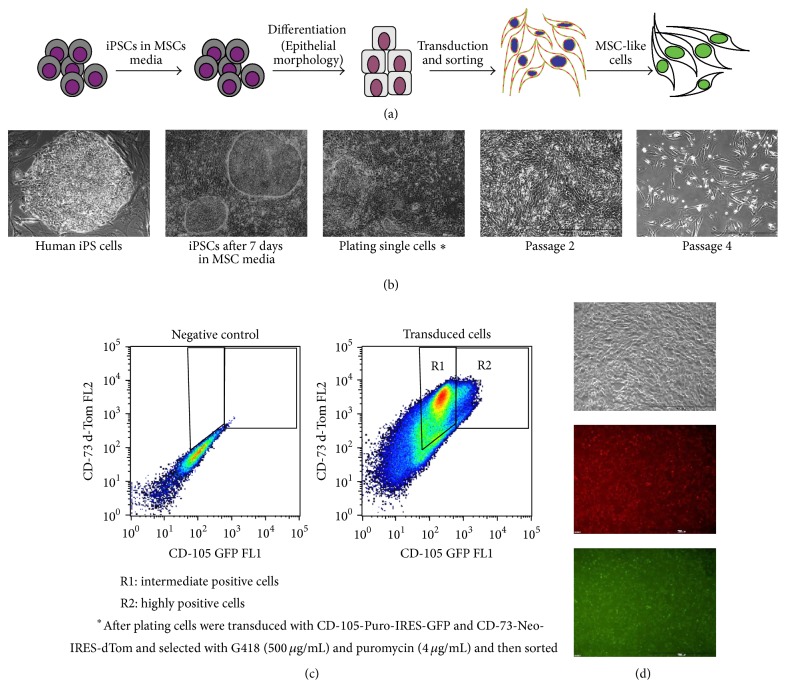
Derivation and enrichment of MSCs from human iPS cells. (a) Schematic stepwise protocol for differentiation and selection of MSC-like cells from human iPS cells. (b) phase-contrast photos demonstrating Epithelial-Mesenchymal transition in cellular morphology. (c) FACS dot blot showing intermediate (R1) and highly (P2) double-positive cells. Highly positive CD-73 and CD-105 (R2) were sorted for upcoming experiments. (d) iPS-MSCs after sorting showed more homogenous mesenchymal morphology expressing GFP/dTom.

The challenge of accessing an appropriate and homogenous source for MSCs with sustained growth kinetics, immunosuppressive potentials, and production of chemokines or growth factors supporting endogenous regeneration led to the question whether homogenously differentiated MSCs could be derived from human induced pluripotent stem cells (iPSCs). To address this question, we used two different sources of somatic cells, liver fibroblast and bone marrow-derived MSCs (BM-MSCs), and reprogrammed these cells into iPSCs. Besides two sources of somatic starting cells, we also compared two slightly different composition of reprograming factor cocktails. One factor combination was comprised of Oct4, Sox2, Klf4, and c-Myc (OSKM) as it was originally described by Shinya Yamanaka and subsequently in multitudinous publications [8–10] and the other combination consisted of Oct4, Sox2, Nanog, and Lin28 (OSNL) as it was described by James Thomson and some further groups [7, 25]. Taken together, we generated fetal liver fibroblast-derived iPSCs with OSKM and OSNL (FLF iPSCs) and bone marrow MSC-derived iPSCs with OSKM (MSC-iPSCs), which were strongly expressing OCT4, SOX2, and SSEA-4 (Figure 1). All iPSC lines were differentiated based on a previously reported differentiation protocol resulting in about 70% CD73^+^/CD105^+^ cells [14], in which we have made some modifications to allow for antibiotic selection and fluorescent reporter-based purification (Figure 2(a)). As expected, Epithelial-Mesenchymal Transition occurred during differentiation giving rise to a heterogeneous population (Figure 2(b)). However, with enrichment of early mesenchymal-like cells, we observed intermediate and highly CD73-dTom/CD105-GFP expressing cells populations (Figure 2(c)). We sorted highly expressing GFP/dTom positive cells and obtained a much more homogenous population (Figure 2(d)). Stimulated by the first description of iPSC-derived MSCs by Lian et al. in 2010 [15], many other groups tried to do direct and spontaneously differentiating iPSCs into MSCs by various means. We consider the use of lentiviral reporter and selection constructs as important tools to monitor the purity of a cell population during differentiation processes and to ensure a high grade of homogeneity within the final cell population. Such selection constructs were recently introduced in other lineages' differentiation protocols [28]. Thus, in the present study a similar vector architecture was applied to select for CD73^+^/CD105^+^ positive iPSC-MSCs. Interestingly, we obtained a high number of CD73^pos^/CD105^intermediate^ iPSC-MSCs (R1: 63.3%) and a smaller fraction of CD73^pos^/CD105^high^ iPSC-MSCs (R2: 6.43%) and we concluded that sorting the less abundant CD73^pos^/CD105^high^ population might provide the most homogenous cell population.

### 3.2. Immunophenotype, Proliferation, and Differentiation Potential of iPSC-MSCs

**Figure 3:**
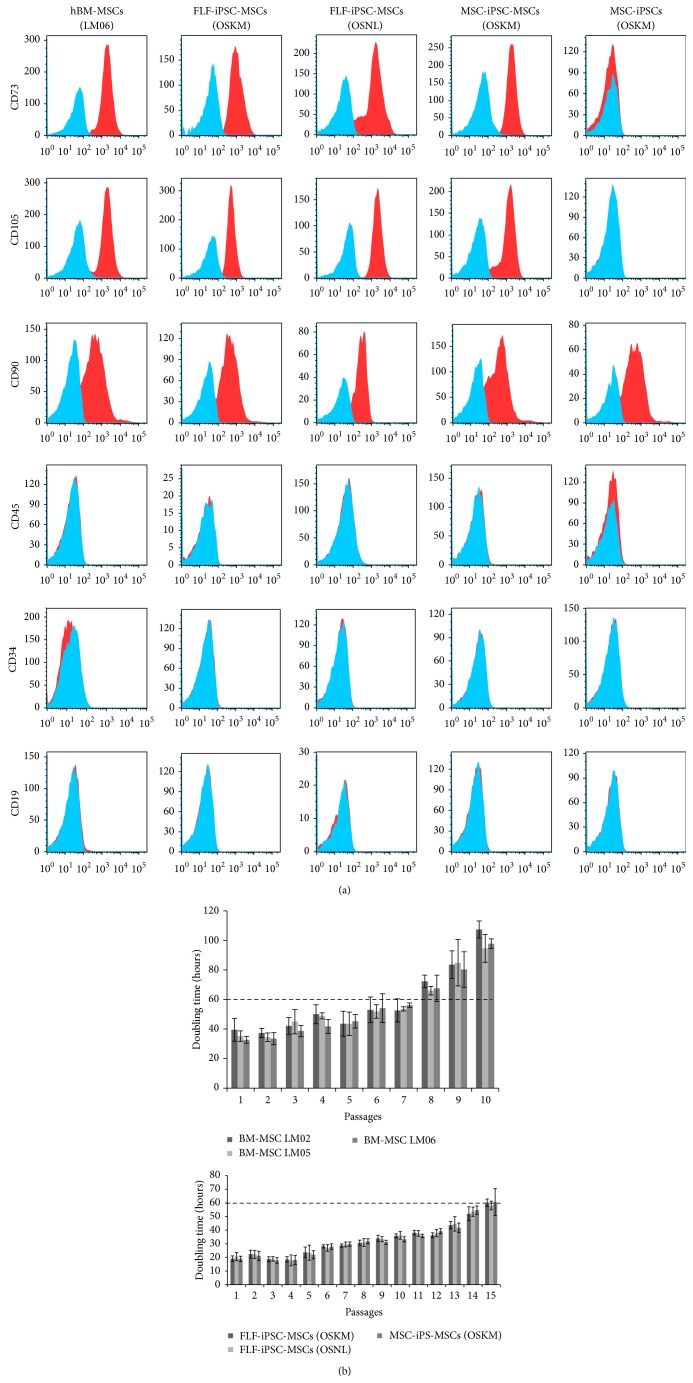
Antigen phenotype, proliferation rate, and functional characterization of hiPSC-MSCs. (a) Immunophenotype of the three hiPS-MSCs lines generated. Representative flow cytometry analysis of hBM-MSCs, FLF-iPSC-MSCs (OSKM), FLF-iPSC-MSCs (OSNL), MSC-iPSC-MSCs (OSKM), and MSC-derived iPSC line MSC-related markers CD73, CD90, and CD105 and hematopoietic CD45, CD34, and CD19 were assessed (solid histogram). (b)* In vitro* cell growth, measured as cumulative population of hiPSC-MSCs and BM-MSCs derived from 3 different donors (LM02, LM05, and LM06).

In order to characterize the iPSC-MSCs according to the International Society of Cell Therapy (ISCT) criteria [29] cell surface marker expression was analyzed by flow cytometry of all three iPSC-MSC lines and BM-MSCs at early passages (passages 3–6). All 3 differentiated and enriched iPSC-MSCs displayed a MSC-like antigen profile that exhibited high CD105, CD73, and CD90 and absence of CD34, CD45, and CD19 expression (Figure 3(a)). Thus, we were able to demonstrate that homogenous populations can be isolated and purified from all three iPSC lines independent to their somatic cell source (fibroblasts or bone marrow MSCs) and method of reprogramming (OSKM or OSNL factor cocktail). Strikingly, the surface marker CD105 and CD73, whose promoter motifs were utilized to express the fluorescent reporter transgenes and antibiotic selection cassettes, were readily detectable in almost 100% of purified cells, indicating the high purity of our enriched iPSC-MSCs. Interestingly, CD90 was positive not only in all iPSC-MSCs lines as well as the BM-MSCs but also in undifferentiated iPSCs (MSC-iPSCs). Furthermore, the hematopoietic surface markers were neither expressed in MSCs nor in iPSCs.

Growth kinetics of iPSC-MSCs demonstrated a greater proliferative capacity when compared with BM-MSCs with shorter doubling times (Figure 3(b)). In our experiments, three independently derived BM-MSCs exhibited doubling times around 36 h in early passages that were prolonged above 60 h around passage 8 and followed by a cessation of proliferation with an apparent senescent phenotype around passage 10 (Figure 3(b)). In contrast, all three iPSC-MSCs exhibited significantly shorter doubling times (around 20 h in early passages). The prolonged doubling time of more than 60 h did not occur before passage 15 and even after 20 passages iPSC-MSCs did not show a senescent phenotype. These results are in line with previously reported data from Sánchez et al., who showed that human embryonic stem cell-derived CD73^+^ and CD90^+^ MSCs had higher proliferation rate than BM-MSCs (ESCs-MSCs~18 doubling compared to BM-MSCs~5 doubling in 30 days of culture) but were similar to umbilical cord derived MSCs (~15 doubling in 30 days) [12]. The more robust proliferation potential of iPSC-MSCs suggests an important advantage over BM-MSCs, whenever repetitive transplantations of the very same MSC batch would be most preferential (for review of this impact on age-related disorders, see [30]). Although the dosing of MSCs perfusion is currently controversially discussed for different disorders, one can assume that an increasing demand of MSCs transplantation may arise in certain disorders. For example, musculoskeletal injuries with high occurrence in seniors [31] may urge for engineered MSCs with higher proliferation capabilities but same functional abilities as BM-MSCs. Thus, iPSC-MSCs may serve as “off the shelf transplant,” which can be provided by blood/stem cell banking institutions and used for several degenerative diseases. Moreover, the higher homogeneity of such well-proliferating, nonsenescent iPSC-MSCs populations suggest a higher safety and efficacy profile and may qualify such cells for more long-term treatment such as inflammatory bowel diseases or during the prevention of graft versus host diseases or transplant rejection in future transplantation settings [12].

**Figure 4:**
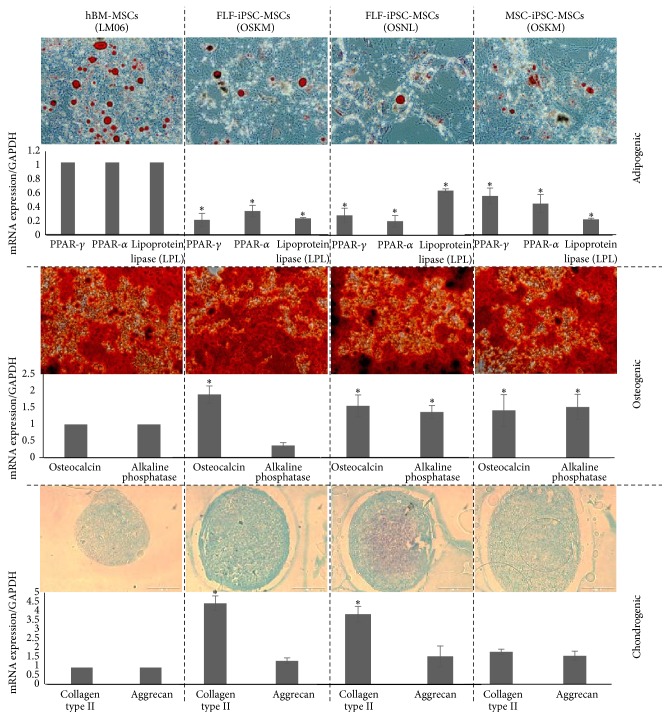
Adipogenic, osteogenic, and chondrogenic differentiation potential of hiPSC-MSCs and BM-MSC (LM06). Oil Red-O staining for lipid formation, alizarin red staining of mineralized deposits, and alcian blue staining for chondrocyte pellet formed by the three iPSC-MSC-like cell lines. mRNA expression level of the relative expression of genes associated with adipogenesis PPAR*γ*, PPAR*α*, and LPL, osteogenesis (osteocalcin and alkaline phosphatase), and chondrogenesis (collagen type II and aggrecan). The data represent the mean expression values normalized to the housekeeping gene GAPDH. *∗*: significance difference with BM-MSCs *p* ≤ 0.05.

Addressing the functional capabilities of iPSC-derived MSCs we applied differentiation protocols towards adipo-, osteo-, or chondrogenic lineages, respectively, and performed cytological staining and RT-PCR to investigate changes in cell morphological and related marker gene expression. Importantly, all 3 iPSC-MSCs could give rise to all of these three lineages. However, in comparison to BM-MSCs the quality and morphology characteristics of the differentiated iPSC-MSCs exhibited slight differences. All iPSC-MSCs were more reluctant to adipogenic differentiation and the respective total numbers of differentiated cells containing lipid droplets were lower than that of BM-MSCs (Figure 4). This observation was confirmed with adipocytes specific mRNA level in which expression levels of PPAR-*α* and PPAR-*γ* were significantly lower in iPSC-MSCs than BM-MSCs. Also LPL expression levels were significantly (*p* ≤ 0.05) lower in FLF-iPSC-MSCs (OKSM) and MSC-iPSC-MSCs (OKSM) compared to BM-MSCs. On the other hand iPSC-MSCs had more affinity to differentiate to osteogenic and chondrogenic lineages. Gradually mineral nodules formation started 1 week earlier in iPSC-MSCs, which were stained positive for alizarin red S. The expression of osteocalcin and alkaline phosphatase was comparable to BM-MSCs. Characterizing chondrogenesis, the respective pellets were stained more strongly with alcian blue and the respective gene expression profiles for collagen II showed higher expression in iPSC-MSCs compared to BM-MSCs. However, Aggrecan was similarly expressed in all three iPSC-MSCs and BM-MSCs. It has been shown that some MSCs for instance from periosteum and synovium were easily capable of differentiating to bone and cartilage, but only a minor population amongst them could give rise to adipocytes [23, 32] and we speculate that such a MSC-related phenotype is resembled by our iPSC-derived MSCs.

### 3.3. Supportive Effects on Long-Term CD34^+^ Cells Maintenance

**Figure 5:**
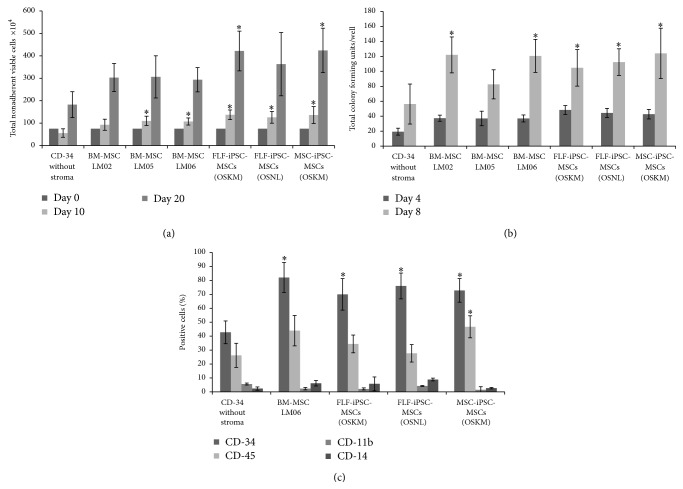
Coculture of CD34^+^ PBMCs with human iPSC-MSCs. (a) Cell layers of iPSC-MSCs and BMSCs were established on 1% gelatin precoated 24-well plates (80% confluent). CD34^+^ PBSCs were applied onto the stromal layers. The cocultures were incubated for 20 days. Nonadherent viable cells were counted at the indicated time points, (b) human CD34^+^ were plated with hiPSC-MSCs in 0.5 mL of methylcellulose media containing human recombinant IL-3, SCF, and Epo. The plates were incubated for 20 days following which progenitors were scored, (c) surface markers expression on CD34^+^ cells after coculture with mesenchymal stromal cells. The results represent the mean (±SD) of three replicates. *∗*: significance difference with CD34 without stroma *p* ≤ 0.05.

The bone marrow niche plays a vital role in preserving hematopoietic progenitors to provide proper amounts of blood cells throughout life [33, 34]. This active microenvironment is fostered by secreted factors of niche-accompanying cells such as MSCs and sinusoidal endothelial cells to support the quiescent state of some of the hematopoietic progenitors [35, 36]. The supportive cellular microenvironment provided by MSCs regulates self-renewal versus differentiation of hematopoietic stem/progenitor cells within the bone marrow [37, 38]. Moreover, based on their secretion of cytokines supportive for hematopoietic cell proliferation, MSC are considered to serve as an excellent cell type for long-term progenitor cell culture purposes [39]. As further indication for the undisturbed functional capabilities of iPSC-MSCs we exploited a coculture system of iPSC-MSCs and CD34^+^ hematopoietic stem/progenitor cells and investigated the total cell numbers, the colony forming capacity, and the homogeneity of CD34^+^ cells. After 10 days, all three iPSC-MSC coculture assays contained significantly (*p* ≤ 0.05) more nonadherent cells compared to CD34^+^ cells cultured without any stroma. Comparing coculture of CD34^+^ cells with iPSC-MSCs and BM-MSCs, we observed a robust (but in our experiments not significant) increase in nonadherent cell numbers for the iPSC-MSCs assays (Figure 5(a)). For OSKM factors-derived iPSC-MSCs (Figure 5(a)) similar results were obtained even at day 20. After replating CD34^+^ cells in MethoCult media for colony forming assays, we observed significantly increased colonies in all iPSC-MSCs and BM-MSCs lines after 4 days of coculture comparing to single CD34^+^ culture. Furthermore, MethoCult culture for 8 days resulted in significantly (*p* ≤ 0.05) higher colony numbers in iPSC-MSCs and in 2 lines of BM-MSCs (Figure 5(b)). This data demonstrates a further important functional aspect and is supported by prior investigations that indicated the supportive nature of MSCs on hematopoiesis by providing a suitable microenvironment for stem/progenitor cells in growing sites [40]. With our data we also provided evidence that significantly higher CD34^+^ cell number maintain their stem cell status on the iPSC-MSCs and BM-MSCs rather than conventional hematopoietic medium (Figure 5(c)). These enhanced proliferation and boosted colony forming abilities could be observed after 8 days of coculture in all iPSC-MSCs lines suggesting that these represent a reliable cell source supporting the long-term culture of hematopoietic stem/progenitor cells. Several publications are in favor of the effects of different feeder layers and coatings for maintenance and expansion of progenitors and somatic cells showing the importance of mimicking* in vivo* conditions and providing similar microenvironment [41–43].

### 3.4. Immunosuppressive Effects of iPSC-MSCs

**Figure 6:**
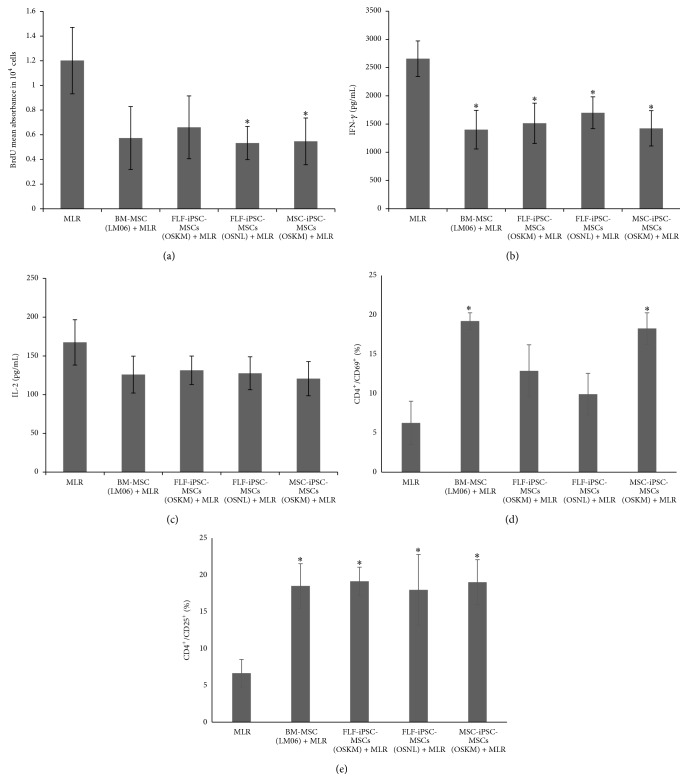
Status of activated CD4^+^ T cells in the presence of hiPSC-MSCs. (a) IFN-*γ* were determined at 48 hours by ELISA. The values are the means ± SD from 3 independent experiments, (b) concentrations of IL-2, and (c) proliferation in MLR/MSC cocultures. MLR cultures were set up in presence or absence of hiPSC-MSCs. BrdU incorporation was significantly lower in MSC-ips-MSCs and FLFiPSC-MSCs (OSNL) in comparison to absence of MSCs. (d) Expression of the T-cell activation markers CD69 and (e) CD25 on CD4^+^ 5 days after stimulation in a 12-well dish in the presence or absence of hiPSC-MSCs. *∗*: significance difference with MLR *p* ≤ 0.05.

In pioneering studies mesenchymal stem cell based approaches were applied for suppressing immune reactions in autoimmune disorders, graft versus host disease (GVHD), or after solid organ transplantation (for review see [44, 45]). During allogeneic cell or organ transplantation, cytotoxic and helper T-lymphocytes get activated and kill the targeted cells or promote rejection of the transplanted organ [46]. Because MSCs can secrete anti-inflammatory molecules to dampen inflammatory reaction [47], one can speculate that iPSC-derived MSCs could also provide a valuable cell source for immunomodulatory therapies (for review see [11]). In order to investigate the immunomodulatory properties of iPSC-MSCs we have used Mixed Lymphocyte Reaction (MLR) to mimic inflammatory reaction by mixing CD4^+^ lymphocytes with healthy donor peripheral blood mononuclear cells (PMNCs) on iPSC-MSCs and BM-MSCs feeder layers, respectively. First, we checked the CD4^+^ lymphocyte proliferation in MLR assay by BrdU incorporation. Human FLF-iPSC-MSCs (OSNL) and hMSC-iPSC-MSCs (OSKM) could significantly (*p* ≤ 0.05) dampen lymphocyte proliferation and we observed a similar decrease in FLF-iPSC-MSCs (OSKM) and BM-MSCs (Figure 6(a)). MSCs are known to exhibit regulatory properties on different kinds of immune cells including T-lymphocytes, but so far it has been insufficiently considered to what extent iPSC-MSCs display this modulating activity. Previously, immune regulatory effects of iPSC-MSCs on Natural Killer (NK) cells have been studied by Giuliani et al., where it was indicated that the NK-cell cytolytic machinery was disrupted by inhibiting NK-cell proliferation and IL-2 activation via expression of different activation markers and ERK1/2 signaling [48]. There is also plenty of evidence that lymphocyte can be suppressed by MSCs secreting anti-inflammatory cytokines in response to proinflammatory stimuli mediated through IL-2 and IFN-*γ* [49, 50]. Therefore we investigated the amount of IFN-*γ* (Figure 6(b)) and IL-2 (Figure 6(c)) in the supernatant of MLR assays from the iPSC-MSC coculture experiments. While we could observe a significant decrease of IFN-*γ* levels in the iPSC-MSC and BM-MSCs coculture experiments, we could only detect a minor reduction of IL-2 levels in the control BM-MSC coculture experiment as well as in the iPSC-MSC experiments. Nevertheless, these results are supporting previous findings that MSCs can dampen inflammatory response via suppressing T-cell proliferation [51] and decreasing proinflammatory cytokines due to nitric oxide production that inhibits Stat-5 phosphorylation in memory and cytotoxic T-cells [4]. Previously it has been reported that MLR coculture with MSCs significantly increases regulatory markers (CD69 and CD25) expressing population in CD4^+^ cells [5, 52–54]. Our results indicated that MSC-iPSC-MSCs and BM-MSCs significantly increased the early T-cell activation marker CD69^+^ population compared to MLR alone (Figure 6(d)). Even if the increase in CD69^+^ population in FLF-iPSC-MSCs did not reach the level of significance, we speculate that these cells have an immunomodulatory impact as well. Moreover all iPSC-MSCs as feeder layer have the ability to significantly increase CD25^+^ population compared to MLR alone (Figure 6(e)), which is in line with previous publications [52, 55].

## 4. Conclusion

Here, we describe a lentiviral selection cassette mediated allowing the enrichment of highly functional human iPSC-derived MSCs from different somatic starting cells. Such iPSC-MSCs exhibited higher proliferation capabilities and similar surface marker compared to bona fide MSCs derived from bone marrow. Moreover, we were able to demonstrate that iPSC-MSCs support the long-term culture of CD34^+^ hematopoietic stem/progenitor cells with undisturbed colony forming abilities. Finally, human iPSC-MSCs also exhibited immunomodulatory function with lowering CD4^+^ T-lymphocyte population, decreasing IFN-*γ* secretion, and increasing regulatory T-cell population. Thus, iPSC-MSCs might be considered as relevant cell resource for future transplantation studies in preclinical models of GVHD and degenerative autoimmune diseases.

## Figures and Tables

**Table 1:** List of antibodies and ELISA kits used in this study.

Antibody/ELISA kit	Company	Dilution	Cat. number
CD-45	BD Bioscience	1 : 100	555485
CD-105	BD Bioscience	1 : 100	562408
CD-34	BD Bioscience	1 : 100	555824
CD-73	BD Bioscience	1 : 100	560847
CD-19	BD Bioscience	1 : 100	555419
CD-90	BD Bioscience	1 : 100	555595
CD-25	BD Bioscience	1 : 100	555431
CD-69	BD Bioscience	1 : 100	555530
CD-4	BD Bioscience	1 : 100	340419
CD-11b	BD Bioscience	1 : 100	557321
CD-14	BD Bioscience	1 : 100	557742
SSEA-4	BD Bioscience	1 : 100	560128
OCT-4	Santa Cruz	1 : 200	SC-5279
SOX-2	Santa Cruz	1 : 200	SC-17320

Human IFN-*γ*	BD Bioscience	—	550612
Human IL-2	BD Bioscience	—	550611

**Table 2:** List of primers used in this study qRT-PCR.

Gene symbol	Sequences 5′-3′, forward	Sequences 5′-3′, reverse	Size (bp)	Annealing temp. (°C)	Accession number
PPAR*γ*	CTAAAGAGCCTGCGAAAG	TGTCTGTCTCCGTCTTCTTG	331	60	NM_015869.4
PPAR*α*	ACTCCGTCTTCTTGATGAT	TGCTATCATTTGCTGTGGAG	215	60	NM_005036.4
LPL	TCAACTGGATGGAGGAG	GGGGCTTCTGCATACTCAAA	169	60	NM_000237.2
COL2a	TCTACCCCAATCCAGCAAAC	GCGTAGGAAGGTCATCTGGA	170	60	NM_033150.2
ACAN	CTGGACAAGTGCTATGCCG	GAAGGAACCGCTGAAATGC	191	60	NM_013227.3
BGLAP (OCN)	GGCAGCGAGGTAGTGAAGAG	CAGCAGAGCGACACCCTAGAC	195	60	NM_199173
ALP	CAACAGGGTAGATTTCTCTTGG	GGTCAGATCCAGAATGTTCC	136	60	NM_000478.4
GAPDH	CTCATTTCCTGGTATGACAACGA	CTTCCTCTTGTGCTCTTGCT	122	60	NM_002046.3
